# Centriolar defects, centrin 1 alterations, and FISH studies in human spermatozoa of a male partner of a couple that produces aneuploid embryos in natural and artificial fertilization

**DOI:** 10.1007/s10815-021-02109-0

**Published:** 2021-02-22

**Authors:** Elena Moretti, Daria Noto, Raffaella Guazzo, Andrea Menchiari, Giuseppe Belmonte, Giulia Collodel

**Affiliations:** 1 Department of Molecular and Developmental Medicine, University of Siena, Siena, Italy; 2 Department of Medical Biotechnologies, University of Siena, Siena, Italy; 3 Department of Business and Law, University of Siena, Siena, Italy

**Keywords:** Aneuploidies, Centrin 1, Centriolar adjunct, FISH, Sperm, TEM

## Abstract

**Purpose:**

To study the potential paternal contribution to aneuploidies in the man of a couple who obtained trisomic embryos with natural and assisted fertilization.

**Methods:**

Semen analysis, immunofluorescence for localization of tubulin and centrin 1, transmission electron microscopy (TEM), and fluorescence in situ hybridization (FISH) analysis for chromosomes 18 and 9 were performed. Sperm of fertile men were used as controls.

**Results:**

The percentages of sperm motility and normal forms were decreased. The percentages of sperm with tail reduced in dimension, headless tails, coiled tails, and altered head-tail junction were significantly higher (*P* < 0.01) in the patient than in controls, whereas the percentage of sperm with a normal centrin 1 localization (two spots in the centriolar area) was significantly reduced (*P* < 0.01) in the patient. Immunofluorescence with anti-tubulin antibody showed that in most of the patient’s sperm connecting pieces (83.00 ± 1.78%), two spots were present, indicating prominent proximal centriole/centriolar adjunct and evident distal centriole, whereas controls’ sperm displayed a single spot, indicating the proximal centriole. The percentage of sperm with two spots was significantly higher (*P* < 0.01) in the patient than in controls. TEM analysis showed that centriolar adjuncts of the patient’s sperm were significantly longer (721.80 ± 122.26 nm) than in controls’ sperm (310.00 ± 64.11 nm; *P* < 0.001). The aneuploidy frequencies of the patient’s sperm, detected by FISH analysis, were increased with respect to controls.

**Conclusion:**

A paternal contribution to sperm aneuploidies cannot be excluded since the patient’s sperm showed altered morphology, immature centriolar adjunct, presence of evident distal centriole, scarce presence of centrin 1, and high aneuploidy frequency.

## Introduction

Embryo aneuploidies can originate from meiotic and mitotic errors [1]. The large maternal contribution to embryo aneuploidies is recognized [2] and, in some way, overshadows the role and clinical relevance of the spermatozoon resulting on a fair consideration of paternal influence to the phenomenon of embryo aneuploidies. On the other hand, the sperm contribution to embryo creation is not only limited to haploid genome. Growing evidence suggests that certain men, even with normal sperm parameters, may have significantly increased levels of sperm aneuploidies [1, 3] that can affect the euploidy of their embryos. In addition, the spermatozoon must provide the oocyte with an activation stimulus, a competent genome, and other factors such as mRNAs and micro RNAs that may regulate transcription or complement the maternal transcriptome within the cleavage stage embryo [4, 5]. Another element that the sperm contributes to the oocyte is the centrosome since human oocyte lacks assembled centrioles [6]. Alterations of centrosome formation and function lead to a variety of situations, such as syngamy failure, improper segregation during embryo mitosis, formation of multiple microtubule-organizing centers with chaotic segregation, and cell division [4, 7, 8]. The centrosome consists of a pair of centrioles associated with proteins that form the microtubule-organizing center of the cell, responsible for correct segregation of the chromosomes during cell division [9]. In the sperm connecting piece, a barrel-shaped proximal centriole (PC), a surrounding pericentriolar material (PCM), and an atypical centriole (distal centriole, DC) composed of splayed microtubules surrounding rods of centriole luminal proteins are present [10]. During spermiogenesis, the centrosome is deeply remodeled by both reduction and enrichment of specific proteins [10, 11]. One of these proteins is the centrin 1, a sperm specific calcium-binding protein involved in the centrosome dynamic during sperm morphogenesis and in the mitotic spindle assembly. In addition, during spermatid maturation, the PC microtubules elongate to build a sort of “mini flagellum” called the centriolar adjunct. Surprisingly, the exact function of this structure is unknown, and there have been very few studies on it. The centriolar adjunct undergoes dynamic changes during sperm maturation, partially or completely disappearing in the mature spermatozoa of different organisms [12]. In human spermatozoa, this structure can be visible as microtubule triplets, doublets, and singlets. It has been suggested that the presence of a centriolar adjunct in human sperm cells can be considered a manifestation of incomplete maturation. At this purpose, disturbances in proximal centriole morphogenesis, in particular the incomplete disassembly of the centriolar adjunct, can be one of the reasons for embryonic development arrest at the zygote stage [6, 12].

In this report, we considered the male partner of a couple who obtained aneuploid progeny with natural and assisted fertilization. After the sperm evaluation and the observation of multiple sperm defects, we performed immunofluorescence analysis to localize tubulin and centrin 1 and an ultrastructural study using transmission electron microscopy (TEM). The sperm chromosomal aneuploidies were assessed by fluorescence in situ hybridization (FISH) analysis for chromosomes 18 and 9.

## Materials and methods

### Patients

This research accounts for the study of spermatozoa from a patient (38 years old) of a couple who attended our laboratory specialized in ultrastructural and immunocytochemical studies of male gametes. The patient and his wife’s lymphocyte karyotypes were normal. The wife (38 years old) demonstrated that physical examination, hysterosalpingogram, and reproductive endocrine evaluation were within standard ranges. The couple does not have children; both partners never tried to conceive previously with other partners. The wife then declared that after a spontaneous abortion at embryo stage (6th week of gestation), she underwent two chorionic villus samplings in as many subsequent pregnancies. In both cases, the result was an individual with a female karyotype suffering from trisomy of chromosome 21.

Subsequently, the couple underwent assisted reproductive technology (ART) treatment in order to carry out the preimplantation genetic testing before embryo transfer. In the ART cycle, 3 embryos were obtained, and after prenatal genetic testing with comparative genomic hybridization array performed in cells obtained with biopsy at the blastocyst stage, they showed trisomy of chromosome 22, chromosome 12, and chromosome 21, respectively. These procedures were performed in laboratories other than ours, and the couple provided us with the results of the analyses.

Both the patient and his wife had no history of consanguinity in their ancestry. The patient showed normal sexual development, medical history, physical examination, and hormone levels. Semen culture did not reveal the presence of genitourinary infections.

Semen samples of three fertile men (aged 32–35 years) were used as controls for immunocytochemistry and TEM analyses. FISH studies were carried out in the spermatozoa of the patient and of a control sample.

The participants signed an informed written consent before participating in this research, accepting that their semen samples and the clinical data they supplied might be used for scientific purposes.

### Semen analysis: light and electron microscopy

Semen samples were collected by masturbation after 4 days of sexual abstinence and examined after liquefaction for 30 min at 37 °C. The analysis of the patient’s semen was repeated three times at intervals of 3 months. Volume, pH, concentration, progressive motility, and percentage of sperm with normal morphology were evaluated according to WHO guidelines [13]. Sperm viability was assessed by staining semen sample with 0.5% eosin Y (CI 45380) in 0.9% aqueous sodium chloride solution. A few minutes after staining, the sample was observed by light microscope and stained (dead) cells and unstained (living) cells were scored.

The chromatin condensation was assessed by the aniline blue (AB, Panreac, Barcellona, Spain) staining as reported in Moretti et al. [14]. The slides were observed and evaluated with a Leitz Aristoplan Microscope (Leica, Wetzlar, Germany), and more than 300 sperm nuclei were scored at × 1000 magnification.

For the ultrastructural analysis, the patient and fertile men’s sperm samples were processed according to the methods described elsewhere [14]. Ultrathin sections, stained with uranyl acetate and lead citrate, were observed and photographed with a TEM-Philips EM208S (Philips Scientifics, Eindhoven, the Netherlands).

We analyzed at least 100 ultrathin sections of each sample in the region where centriole adjunct can be present, both in the patient and controls’ spermatozoa. Only longitudinal sections in which PC had a parallel orientation to the plan of the section were measured and compared (20 for the patient and 10 for the controls). The length of centriolar adjuncts was measured with Image J open source image processing program (NIH; USA).

### Immunocytochemistry

The sperm samples of the patient and of the three fertile men were washed in phosphate buffer saline (PBS) twice, smeared on glass slides, air dried, and processed as reported elsewhere [15]. After fixation (methanol 20 min and acetone 5 min at -20 °C) and an incubation in blocking solution (PBS-bovine serum albumin (BSA) 1%, normal goat serum (NGS) 5%), the slides were incubated overnight at 4 °C with mouse monoclonal anti-β-tubulin antibody (Sigma, Chemical, St Louis, MO) diluted at 1:100 and mouse monoclonal anti-CETN1 antibody (Sigma, Chemical, St Louis, MO) diluted at 1:20. Detection was performed with a goat anti-mouse IgG-Alexa Fluor 568 (Invitrogen, Thermo Fisher Scientific, Carlsbad, CA, USA) diluted at 1:100. Incubation in primary antibodies was omitted in control samples. After incubation, the slides were treated with 4′,6-diamidino-2-fenilindole (DAPI, Vysis, Downers Grove, IL) solution and observed with Leica DMI 6000 (Leica Microsystems, Germany) fluorescence microscope. The images were acquired with a Leica AF6500 Integrated System for Imaging and Analysis (Leica Microsystem, Germany). The experiments were repeated three times; for each experiment, at least two slides for each subject were evaluated and 300 sperm examined.

### FISH analysis

Samples were processed for FISH using the methods described in Sarrate and Anton [16]. After liquefaction, the semen samples were washed with pre-warmed KCl 0.075 M; then, they were processed with Carnoy’s solution (3:1 methanol: acetic acid) and subsequent centrifugation as many times as necessary to obtain a white pellet. Finally, the samples were used to smear slides, which were stored at -20 °C.

The slides were placed in 2x saline-sodium citrate solution (2xSSC) at 37 °C for 3 min twice, transferred through a series of ethanol washes (70%, 90%, and 100%), and air-dried. Chromatin decondensation was carried out with dithiothreitol solution (1,4-dithiothreitol 5 mM, 1% Triton X-100, 2-Amino-2-[hydroxymethyl]-1,3-propanediol 50 mM) at 37 °C for 8 min, and then the slides were washed in 2xSSC for 3 min. Samples were dehydrated in an ascending alcohol series (70%, 90%, and 100%) and air-dried before denaturation. Sperm DNA was denatured by incubating the slides in formamide solution (70% formamide/2xSSC) at 73 °C for 5 min, transferred in ethanol solutions (70%, 85%, and 100%), and dried out at room temperature. A mix of α-satellite DNA probes (CEP, Chromosome Enumeration Probes, Vysis, IL, USA) for chromosomes 9 (SpectrumOrange) and 18 (SpectrumAqua) was added, and the slides were kept at 37 °C o/n. Then, the slides were washed with 73 °C pre-warmed 0.4xSSC/0.3% NP-40 and with 2xSSC/0.1% NP-40 at room temperature for 1 min to eliminate the unspecific hybridization signals. To stain the nuclei, DAPI (Vysis, Downers Grove, IL) solution was added. The slides were observed, and the images were acquired using a Leica AF6500 Integrated System for Imaging and Analysis (Leica Microsystem, Germany) equipped with single band pass filters for TRITC, FITC, and DAPI. Sperm nuclei were scored according to published criteria [17]. Only the slides with more than 98% of hybridization efficiency were considered. Intact and non-overlapped sperm nuclei were scored. A nucleus was considered disomic if it showed two fluorescent spots of the same color, comparable in size, shape, and intensity, and located within the same sperm head at least one domain apart. Diploidy was recognized by the presence of two double fluorescent spots following the above criteria. All samples were analyzed by a highly trained examiner who scored 5346 spermatozoa of the patient and 5503 of the control.

### Statistical analysis

Statistical analysis was performed with SPSS 17.0 for Windows (SPSS Inc., Chicago, IL, USA).

The Kolmogorov-Smirnov test was used to verify the normality in the distribution of the variables. The variable related to the centriolar adjunct length was normally distributed; the others were non-normally distributed. To compare the length of centriolar adjuncts between the patient and controls’ sperm, Student’s *t* test was used. Mann-Whitney test was applied to compare the non-normally distributed variables measured in the patient and controls’ sperm. The values were expressed as mean and standard deviation. *P* < 0.05 was considered significant.

## Results

The analysis of the three semen samples showed a normal semen volume (range: 2.0–3.5 mL) and pH (7.2–7.4). Sperm concentration ranged from 20 × 10^6^ to 35.5 × 10^6^ sperm/mL (corresponding to the values included between 5th and 25th centile, [13]), sperm progressive motility ranged from 18 to 25% (< 2.5 centile, [13]), and sperm vitality was normal (70–75%, 10th–50th centile [13]). The sperm morphology appeared compromised at light microscopy level: In the three samples analyzed, the percentages of sperm with normal morphology were between 3 and 4% (≤ 5th centile [13]). The sperm chromatin immaturity assayed with aniline blue (AB) staining ranged from 50 to 62%. The frequent alterations accounted for an irregular head-tail attachment and the presence of headless tails concomitant with sperm showing coiled tails. The problem in the connecting piece negatively influenced the progressive motility, and in some spermatozoa, the head motion was abnormal. For this reason, we decided to explore the spermatozoa of the patient with immunocytochemistry and TEM. To compare the results, spermatozoa from three fertile men were used. The sperm parameters of control samples were > 50th centile of the reference value reported in WHO guidelines [13].

**Fig. 1:**
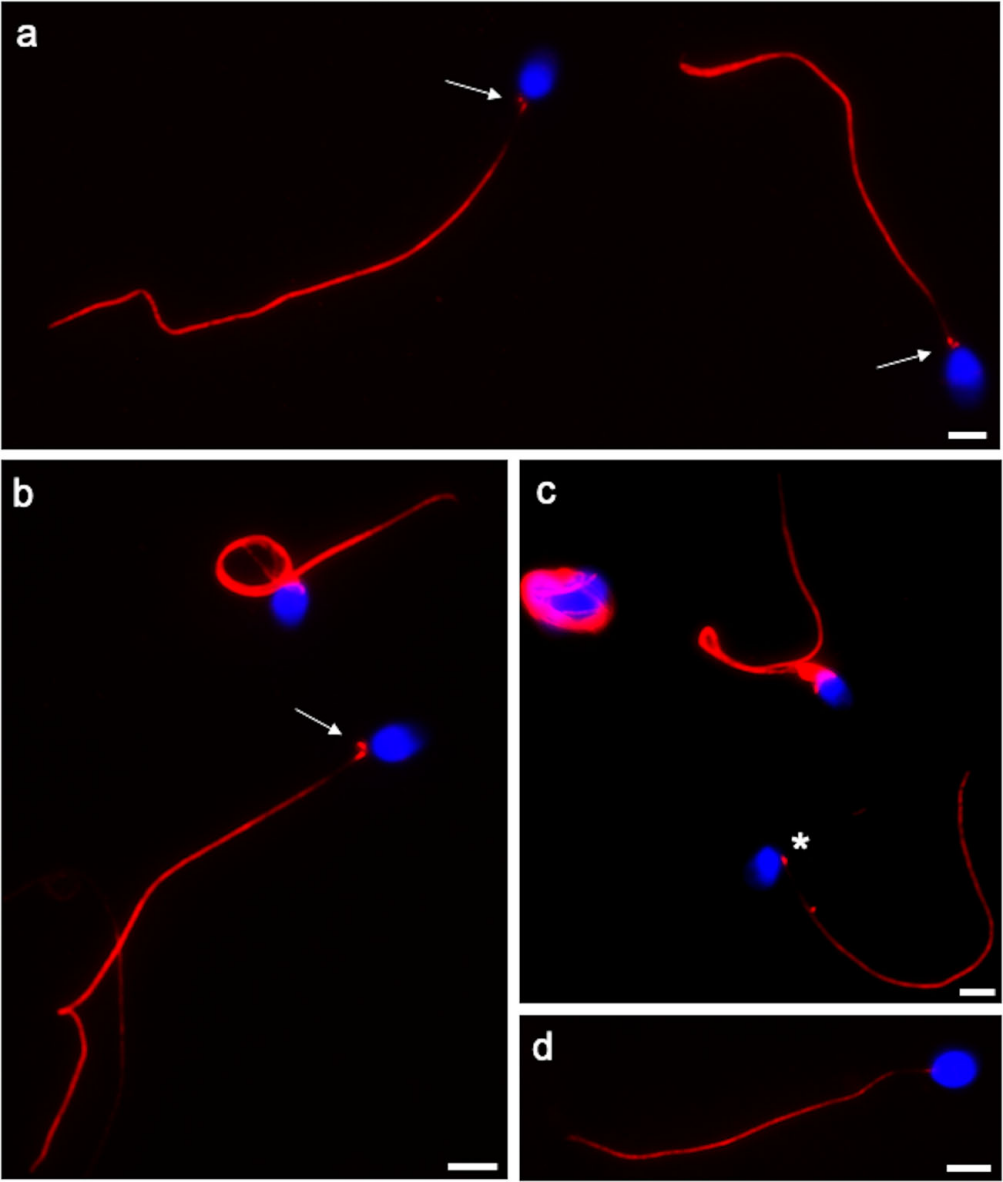
UV micrographs of the patient’s sperm (a, b, c) and control’s sperm (d) treated with a monoclonal antibody anti-tubulin. The patient’s sperm show normal length flagella and coiled flagella. The two spots (arrows) located in the connecting piece probably represent a prominent proximal centriole/centriolar adjunct (the spot closer to the nucleus and perpendicular to the tail midline) and an evident distal centriole. In figure c a spermatozoon with an evidently altered head-tail junction (asterisk) is shown. A normal spermatozoon of a fertile man shows a tiny spot representing the proximal centriole (d). The nuclei are stained with DAPI. Bars: 6 μm

Immunolocalization of tubulin enabled to highlight the patient and controls’ sperm flagella. The results are reported in Table 1 and in Fig. 1.

**Table 1:** Morphology of sperm flagella of the patient and controls evaluated after tubulin immunolocalization

	Normal %	Reduced tail dimension %	Headless sperm tail %	Altered head tail junction %	Coiled tail %
Patient	30.67 ± 2.94*	7.66 ± 1.86*	19.00 ± 3.09*	18.16 ± 1.94*	24.83 ± 2.31*
Controls	81.66 ± 2.58	3.16 ± 1.47	1.8 ± 0.98	0.66 ± 0.51	12.66 ± 1.75

*P < 0.01

In the patient, the percentage of sperm with normal flagella (Table 1, Fig. 1a,b) was significantly lower than in controls (*P* < 0.01; Table 1, Fig. 1d). On the contrary, the percentages of sperm with tail reduced in dimension, headless tails (Table 1), coiled tails (Table 1, Fig. 1b,c), and altered head-tail junction (Table 1, Fig. 1c) were significantly higher (*P* < 0.01) in the patient than in controls. The labeling with anti-tubulin antibody enabled to observe that in the connecting piece of normal sperm a single spot, indicating the proximal centriole, was present (Table 2, Fig. 1d), whereas in most of the patient’s sperm (83.00 ± 1.78%), two evident spots were highlighted (Table 2, Fig. 1a,b). The percentage of sperm with two spots was significantly higher (*P* < 0.01) in the patient than in controls.

**Table 2:** Labeling with anti-tubulin antibody in the connecting piece area

	1 spot%	2 spots%	0 spots%
Patient	11.33 ± 1.96*	83.00 ± 1.78*	5.66 ± 3.44
Controls	80.16 ± 2.78	15.00 ± 4.47	4.83 ± 2.99

*P < 0.01

**Fig. 2:**
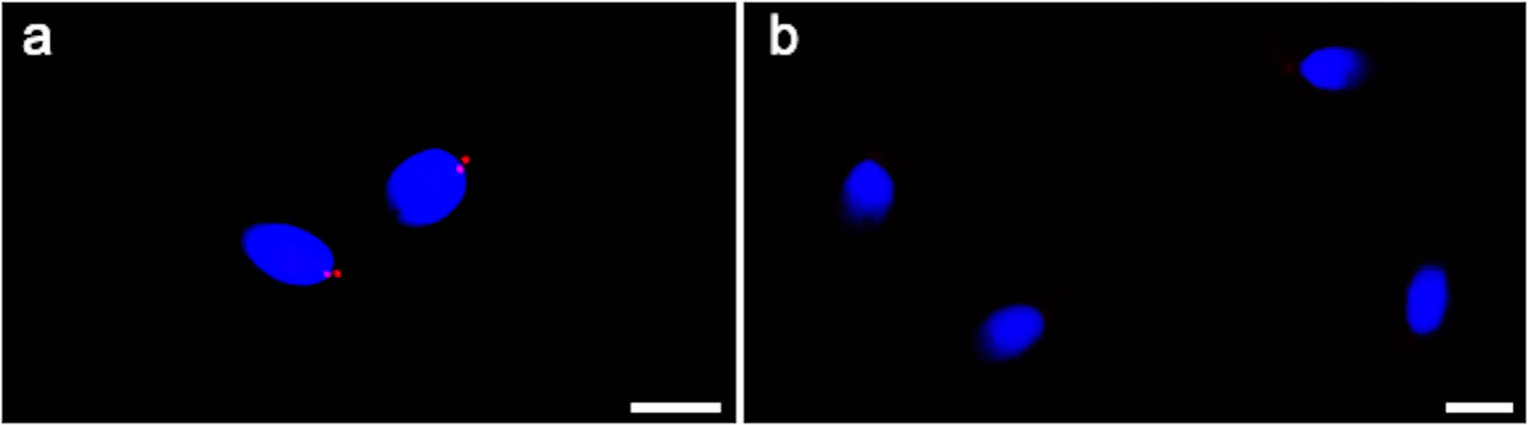
UV micrographs of control’s sperm (a) and the patient’s sperm (b) treated with a monoclonal antibody anti-centrin 1. The normal labelling of centrin 1 (a) consists in two spots localized at the base of the head in the centriolar area. Spermatozoa of the patient showed an almost totally lack of labelling (b). The nuclei are stained with DAPI. Bars: 6 μm

In 91 ± 1.86 % of controls’ spermatozoa, immunofluorescent staining for centrin 1 appeared as two distinct spots located at the base of the nucleus (Table 3, Fig. 2a) in correspondence of the centrosomal region; this value was significantly higher (*P* < 0.01) than in the patient’s sperm (14.66 ± 2.33%). The 79 ± 1.50% of patient’s spermatozoa were negative for the labeling (Table 3, Fig. 2b), and this value was significantly higher than in controls.

**Table 3:** Labeling of spermatozoa with anti-centrin 1 antibody.

	2 spots%	1 spot%	0 spot%
Patient	14.66 ± 2.33*	5.66 ± 2.05	79 ± 1.50*
Controls	91 ± 1.86	4.66 ± 1.36	3.66 ± 1.96

*P < 0.01

**Fig. 3:**
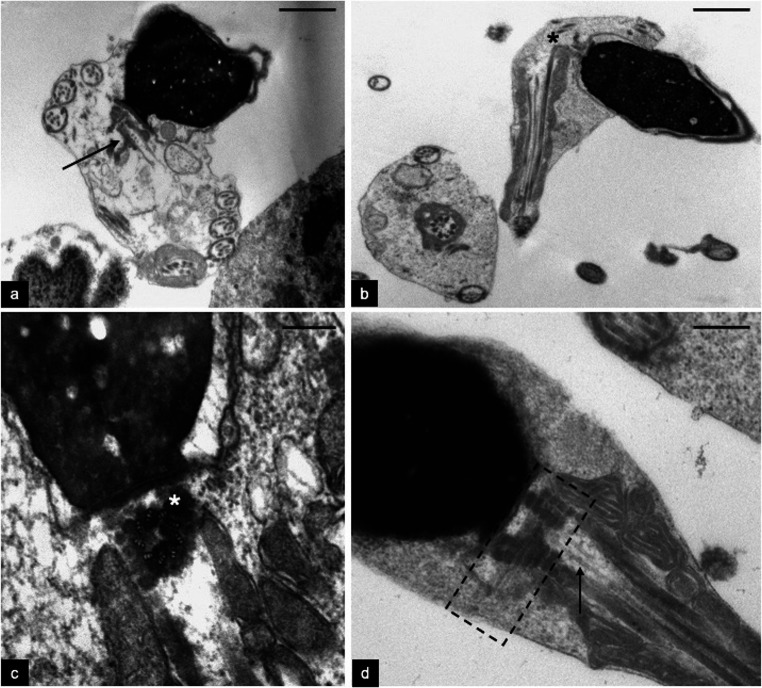
TEM micrographs of longitudinal sections of the patient’s spermatozoa. A centriolar adjunct increased in length (arrow) and located in a wide cytoplasmic residue with coiled tail is shown in figure a. A spermatozoon with bent tail and broken head-tail junction (asterisk) is show in figure b. The asterisk in figure c indicates a perfect longitudinal section of a proximal centriole. In figure d the dotted outline follows the extension of a centriolar adjunct increased in length. The arrow indicates the area where some microtubules of distal centriole are visible. Bars: 2 μm (a,b); 200 nm (c, d)

TEM analysis was used to better understand the alterations of spermatozoa. With a low percentage of morphologically normal spermatozoa, the most frequent alterations regarded the chromatin immaturity, characterized by altered packaging and granular texture (Fig. 3a,b), the presence of cytoplasmic residues with coiled tails (Fig. 3a,b), and particularly alterations in the connecting piece. In fact, we observed sperm with bent tails (Fig. 3b) as we detected at light microscopy level. The PC was well structured (Fig. 3c) in many observed sections.

**Fig. 4:**
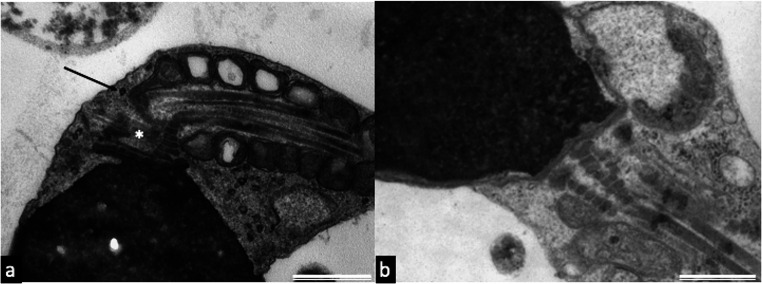
TEM micrographs of longitudinal sections of the patient (a) and controls’ spermatozoa (b). In figure a, the connecting piece is characterized by a centriolar adjunct increased in length (asterisk) and a concomitant defect in head-tail attachment (arrow). Figure b shows a section of a spermatozoon without centriolar adjunct of control sample. The section plausibly represents the area where the centriole adjunct can be located, if present. Bars: 700 nm (a), 400 nm (b)

Interestingly, in the patient’s sperm, we observed centriolar adjuncts significantly increased in length (721.80 ± 122.26 nm, 20 measurements in perfect longitudinal sections) with respect to those observed in the spermatozoa of fertile controls (*P* < 0.001; 310.00 ± 64.11, 10 measurements in perfect longitudinal sections). The presence and increased length of centriolar adjunct are reputed to be a marker of incomplete sperm maturation (Figs. 3a,d and 4a). In Fig. 4a, a long centriolar adjunct emerges from the PC of a spermatozoon with an altered head-tail junction, suggesting that the presence of this structure may weaken the connecting region.

In most of the controls’ spermatozoa, the centriolar adjuncts were not evident (Fig. 4b). The TEM results obtained confirmed the observations of immunofluorescence studies.

**Fig. 5:**
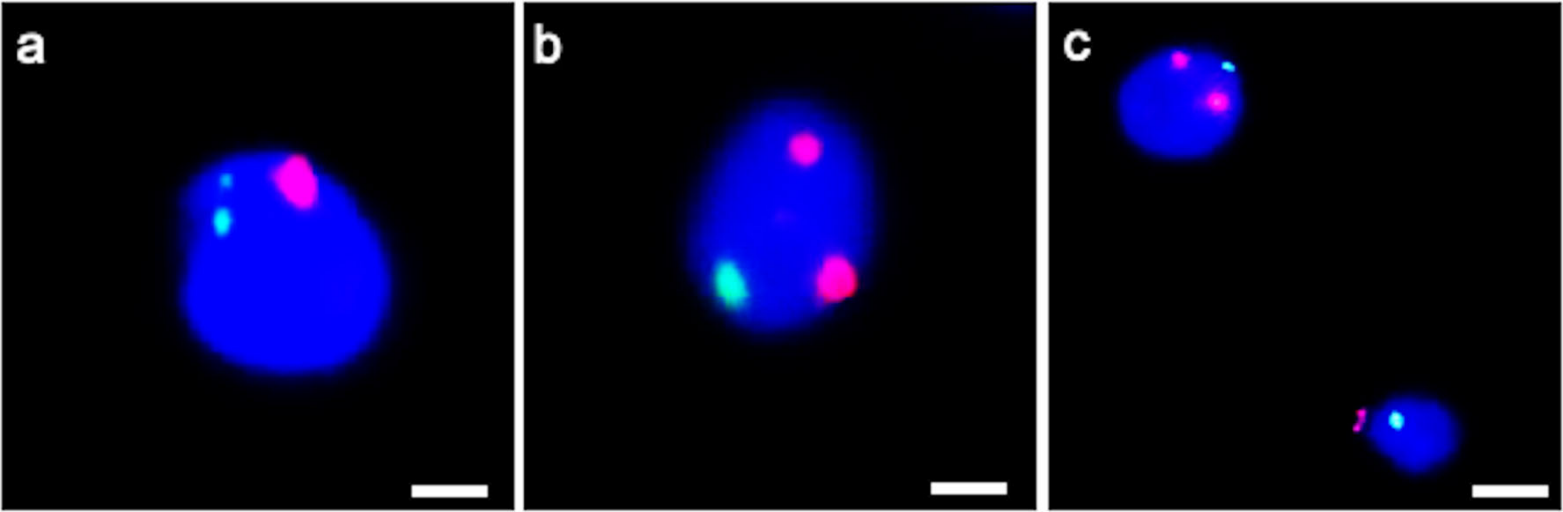
UV micrographs of the patient’s spermatozoa hybridized with probes for chromosome 18 (green) and 9 (red). Disomy of chromosome 18 (a) and chromosome 9 (b, c) are shown. Sperm nuclei are stained with DAPI (blue). Bars: 2 μm (a, b); 4 μm (c)

Regarding FISH analysis, an increase in the percentages of aneuploidies of chromosome 18 (Fig. 5a) and chromosome 9 were observed (Fig. 5b,c) with respect to those observed in the sperm of a fertile control (Table 4).

**Table 4:** Mean frequencies of disomies and diploidies of chromosomes 18 and 9 in the sperm of patient and control

	Diploidy frequency %	Disomy frequency %	
		18	9
Patient	1.04	1.2	1.6
Fertile control	0.32	0.13	0.08

## Discussion

The idea to describe the present case derives from the fact that the couple produced only aneuploid embryos, either with natural or assisted fertilization, and the analysis of spermatozoa showed peculiar defects, in particular in the connecting piece region, despite the fertility of the patient. It is well known that over the last decades, a severe decline in semen quality was observed [18, 19]. Although semen analysis represents a rough estimation of male fertility, it is the only first level exam to test the seminal status of a man. In fact, impaired semen parameters alone cannot be used to predict fertility as these men have still a chance of being fertile, unless the man is affected by azoospermia, necrozoospermia, or systematic sperm defects [20]. This also applies to the case described in this study since we are facing a situation in which the man is fertile but his spermatozoa are mostly defective.

Immunocytochemical and TEM studies enable the analysis of the distribution of several structures, often altered in pathological situations [15, 21–23]. The patient’s spermatozoa, even those with motility, showed defects of the connecting piece region, the area where the sperm centrosome and centrioles are located. Mutations affecting the centriole and centriolar proteins demonstrated the crucial role of centriole in connecting the head and tail, in both humans and mice sperm [24].

By immunocytochemistry with antibody anti-tubulin, spermatozoa showed the presence of two spots in the connecting piece region never observed in such a high percentage in the sperm of controls. The two spots probably represent a prominent PC/centriolar adjunct and an evident DC, typical condition of spermatid stage indicating immaturity of the neck region [10, 11]. Recently, Fishman et al. [10] demonstrated that in mature spermatozoa, an atypical DC is present in addition to the PC. To be atypical, the DC must undergo structural modifications and elimination, reduction, or rarely enrichment of centrosomal proteins, depending on the type of protein. The atypical DC is able to recruit pericentriolar materials, to form a new functioning centriole and participate to the mitotic spindle assembly during embryo mitotic divisions, as reported also in insect spermatozoa [25].

By TEM analysis, the sperm PC can be evaluated in perfect cross sections and the centriolar adjunct in sections where the PC has parallel orientation to the section plane. It is even more difficult to find longitudinal and cross section of DC that probably can be better highlighted by electron microscopy with high-pressure freezing [10]. However, with conventional TEM analysis, we found that the complex PC and centriolar adjunct of the patient’s spermatozoa was significantly increased in length with respect to that of the sperm of controls. During human spermiogenesis, at spermatid stage, the centriolar adjunct is assembled and seems to play a role as the microtubular organization center of the manchette, at least in animal models [26]; then during human sperm maturation, this structure is reduced. The persistence of centriolar adjunct may represent an incomplete maturation of the sperm neck area [11]. The literature on this topic is scant; however, Garanina et al. [12] observed that PC/centriolar adjuncts were increased in length in the spermatozoa of two patients with unexplained infertility (patient 1, 1059 ± 100 nm, and patient 2, 769 ± 160 nm). They suggested that the increased length of this structure can affect fertility and might be responsible for the zygote arrest. In our study, the dimensions of centriolar adjunct in the patient’s spermatozoa, despite natural fertility, is similar to that observed in patient 2 analyzed by Garanina et al. [12] and close to the higher value measured in sperm from fertile individuals (300–699 nm [12]).

The alteration of the head-tail junction in the spermatozoa studied was also confirmed by the immunocytochemical study of centrin 1, one of the proteins needed for DC maturation [10], scarce, and often absent in all the sperm analyzed. At this purpose, the sperm head-tail detachment and consequent sterility were observed in a mouse model gene deleted for centrin 1 [27], suggesting a relevant role of this protein in the correct interaction between basal body and sperm nuclear membrane. No information on eventual aneuploidies in the spermatozoa of this animal model has been reported. In normal sperm, centrin 1 is visible as two distinct spots in the head-tail junction and can show defective pattern of distribution in altered spermatozoa [10, 14, 15, 28–30]. Centrin 1 is a protein almost exclusively expressed in spermatozoa and seems to be lost inside the oocyte after fertilization. These observations suggested that the zygotic centrosomal apparatus can function without centrin, which seems degraded by ubiquitin-proteasome system [28]; alternatively, centrin could be functionally and immunologically hidden within the oocyte by forming complexes with other proteins [31]. Our results agree with these observations and indicate that centrin 1 does not seem to have a role in the fertilization, since our patient is fertile with spermatozoa almost devoid of centrin 1; however, the couple produces aneuploidy embryos. These aneuploidies could be plausibly due to an incorrect meiosis of the oocyte [2]; however, it is possible to hypothesize a crucial, or at least concomitant, role of spermatozoon considering the function of sperm centrosome in embryo division.

Spermatozoa contribute to reduce embryo quality from numerous points of view: the possible presence of sperm aneuploidies, abnormal chromatin structure, DNA fragmentation, role of protamines and histones, Y chromosome microdeletions [5], and altered formation and function of the centrosome. By injecting sperm with mutations of CEP135, a centriole-associated protein, Sha et al. [32] suggested that sperm centrioles are not essential for zygote cleavage but for the subsequent development of the early embryo.

During the last decades, FISH was mainly applied to evaluate human sperm aneuploidies, and the results demonstrated an increase in the frequency of sperm disomies and diploidies in men with spermatogenetic impairment [1, 33, 34]. However, Ramasamy et al. [35] demonstrated that up to 45% of men with recurrent pregnancy loss (RPL) and normal sperm parameters showed increased sperm aneuploidies, suggesting a potential contribution of sperm to embryo aneuploidy, as previously demonstrated also by other authors [36]. FISH analysis revealed a higher frequency of aneuploidies in the patient’s spermatozoa than in the fertile control in line with the data reported in other studies [37, 38]. However, this increase in diploidy and disomy is most likely not responsible for spontaneous abortion and embryo aneuploidy. All the sperm defects observed can be dependent from centriole alterations according to the centriolar roles clearly revised by Avidor-Reiss et al. [24]: Centrioles play a crucial role in regulating early spermatogenic cell divisions, structuring the sperm flagellum, connecting sperm head and tail, controlling sperm tail movement, and organizing the cytoskeleton of the zygote post-fertilization. In particular, since the centriole regulates cell division and participates in spindle assembly and positioning, alterations of this structure can potentially cause chromosomal nondisjunction both during spermatogenesis leading to sperm aneuploidies and during embryo cleavage leading to embryo aneuploidy and recurrent pregnancy loss.

These results underline the importance of implementing investigations on the connecting piece of human spermatozoa and the study of the centriolar adjunct, a structure still little explored. This can help identify defects affecting the sperm centrioles and characterize their impact on male infertility and embryo quality.

## Data Availability

Data generated and analyzed during this study are included in this published article and are also available from the corresponding author.
